# Neurochemical Mechanisms Underlying Alcohol Withdrawal

**Published:** 1998

**Authors:** John Littleton

**Affiliations:** John Littleton, MD, Ph.D., is a professor in the Department of Pharmacology, University of Kentucky, Lexington, Kentucky

**Keywords:** AOD withdrawal syndrome, neurochemistry, biochemical mechanism, AOD tolerance, brain, homeostasis, biological AOD dependence, biological AOD use, disorder theory, biological adaptation, animal model, GABA receptors, glutamate receptors, calcium channel, proteins, detoxification, brain damage, disease severity, AODD (alcohol and other drug dependence) relapse, literature review

## Abstract

More than 50 years ago, C.K. Himmelsbach first suggested that physiological mechanisms responsible for maintaining a stable state of equilibrium (i.e., homeostasis) in the patient’s body and brain are responsible for drug tolerance and the drug withdrawal syndrome. In the latter case, he suggested that the absence of the drug leaves these same homeostatic mechanisms exposed, leading to the withdrawal syndrome. This theory provides the framework for a majority of neurochemical investigations of the adaptations that occur in alcohol dependence and how these adaptations may precipitate withdrawal. This article examines the Himmelsbach theory and its application to alcohol withdrawal; reviews the animal models being used to study withdrawal; and looks at the postulated neuroadaptations in three systems—the gamma-aminobutyric acid (GABA) neurotransmitter system, the glutamate neurotransmitter system, and the calcium channel system that regulates various processes inside neurons. The role of these neuroadaptations in withdrawal and the clinical implications of this research also are considered.

During the past 25 years researchers have made rapid progress in understanding the chemical activities that occur in the nervous system (i.e., the neurochemistry) during alcohol withdrawal (AW), primarily from experiments using animal models of alcohol dependence (Samson and Harris 1992). At the same time, advances have been made in the clinical treatment of AW, making detoxification a routine procedure that no longer is life-threatening (Romach and Sellers 1991). For the most part, however, these treatment advances have evolved independently from the developments in neurochemical understanding. The reasons for this independence are complex, and they include the limited clinical relevance of some of the basic science models used to study withdrawal neurochemistry as well as a reluctance on the part of clinicians to consider new treatments. No reason exists to criticize either scientists or clinicians for this situation; from the basic science viewpoint, it was necessary to understand the neurochemical processes in relatively simple models first, and from the clinicians’ viewpoint, it made no sense to change withdrawal treatments (e.g., the use of benzodiazepines) that had proven successful.

Now may be the right time to rethink our goals for both research and treatment of AW, however. This article reviews current concepts and developments in basic research on the neurochemical mechanisms underlying AW. The article also considers some of the reasons why advances in basic research have not yet been translated into therapeutic gains and suggests areas for future improvement.

## Neurochemical Concepts of Physiological Dependence

It has been said that Darwin’s theory of natural selection is the “single best idea” in biology. A candidate for a similar tribute in drug-dependence research is the suggestion made by C.K. Himmelsbach more than 50 years ago regarding the relation of tolerance, dependence, and withdrawal (Himmelsbach 1941). Based on his observations of morphine-dependent patients, Himmelsbach suggested that physiological mechanisms which maintain a stable state of equilibrium (i.e., homeostasis) in the patient’s body and brain are responsible for drug tolerance, but absence of the drug exposes these same homeostatic mechanisms and leads to the withdrawal syndrome. Clinically, this theory provides the framework for the modern category of “physiological dependence” and its current diagnostic criteria—the presence of tolerance and/or a specific withdrawal syndrome (American Psychological Association 1994). Himmelsbach’s concept has dominated almost all neurochemical explanations for withdrawal from alcohol and other drugs since it was first proposed.

**Figure 1:**
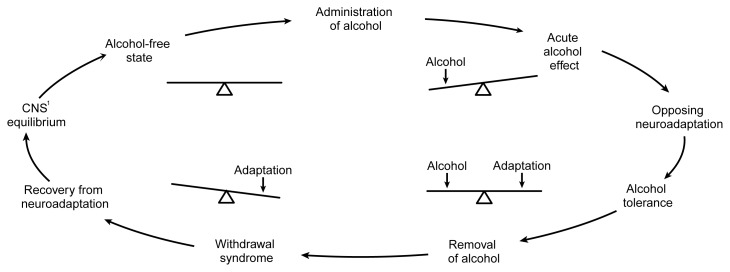
Pictorial representation of the Himmelsbach hypothesis as it applies to alcohol use. The balanced seesaw on the upper left side of the cycle represents brain neurochemistry in an alcohol-free state (i.e., before the brain has been exposed to alcohol). Consuming alcohol initially unbalances brain chemistry to produce the acute effects associated with alcohol use (e.g., sedation and incoordination). The brain then responds to this disruption by inducing an opposing chemical adaptation that tends to restore the neurochemical balance. At this stage, the effects of a given dose of alcohol are diminished (i.e., tolerance exists). If alcohol is removed, the adaptation is exposed, unbalancing the brain’s neurochemistry in the opposite direction. The result is a withdrawal syndrome that includes signs and symptoms (e.g., agitation and seizures) that are opposite to alcohol’s initial effects. These disturbances will continue until the adaptation can be removed from the brain (or until alcohol is consumed again), restoring equilibrium. ^1^CNS = central nevous system.

At the neurochemical level, Himmelsbach’s idea can be expressed as a seesaw (see figure 1). The brain is in a balanced state before alcohol exposure. When a person consumes alcohol, its acute effect (which often is the effect the person is seeking) is to unbalance the neurochemical equilibrium of the brain, thereby tilting the seesaw. If the alcohol exposure continues, the brain institutes an opposing adaptation (i.e., neuroadaptation) to balance the effect of the alcohol and restore neurochemical equilibrium. In other words, the brain develops tolerance for the drug. However, the brain is also in a state of physiological dependence at this point: When alcohol exposure ceases, the new neuroadaptation now unbalances the brain’s neurochemistry. And, like tilting the seesaw to the opposite side, the unbalanced adaptation produces a functional effect on the brain (i.e., the withdrawal syndrome) that is opposite from the effects originally produced by alcohol. Thus, in Himmelsbach’s scheme, the cause of both tolerance and the withdrawal syndrome is the same: an adaptive neurochemical response designed to oppose alcohol’s acute neurochemical effects. It should be emphasized, however, that some other types of tolerance mechanisms can develop without necessarily causing a withdrawal syndrome and, as discussed later, this simple scheme does not take into account what may happen after repeated episodes of alcohol administration and withdrawal.

Himmelsbach’s idea has provided a much-needed framework for investigations of the neurochemical adaptations that occur in alcohol dependence and the way in which these adaptations may precipitate withdrawal. Although the adoption of this framework has been largely beneficial, it also has been restrictive in many ways, not least because it has been interpreted to imply that all the elements involved in tolerance, dependence, and withdrawal should be found within a single neurochemical system. For example, if alcohol is found to inhibit (i.e., reduce the activity of ) a particular chemical messenger (i.e., neurotransmitter^1^) system in the brain, then the subsequent adaptation is predicted to increase activity in the same neurotransmitter system. This increase in neurochemical activity should counterbalance alcohol’s effects, causing tolerance when alcohol is still present and a withdrawal syndrome when it is removed. An alteration of this type (i.e., within the same system) is referred to as a “homologous” adaptation. One of the complexities of the human brain, however, is that an effect on one transmitter system may be counterbalanced by an adaptation in a totally different system (i.e., a heterologous adaptation) (Littleton and Little 1994). Such heterologous adaptations are much more difficult to discern. However, it may be these adaptations—rather than the simpler homologous adaptations—that are responsible for causing tolerance, dependence, and withdrawal.

To complicate matters further, homologous and heterologous adaptations are not mutually exclusive, and both may occur in the same neurons. Alternatively, each type of adaptation may occur in different parts of the brain in response to the same drug. In addition, neurochemical adaptations appear to build up at different rates in a hierarchical process. For example, when alcohol inhibits a specific neurotransmitter system, the system may first adapt by increasing the amount of the chemical messenger released. If the presence of alcohol persists, the system’s next adaptation might be to amplify the effect of the neurotransmitter on its targets (i.e., receptors) in the brain. The adaptation of target cells can be accomplished in several ways. Short-term chemical modifications of receptor proteins on the target neurons^2^ can increase (or decrease) the neurotransmitter’s effects. To use an example from another drug of dependence, the immediate effects of nicotine on nerves are limited because “nicotinic” receptors for the transmitter acetylcholine become desensitized (both to nicotine and acetylcholine). Desensitization of those receptor proteins is caused by short-lived modifications in their structure. Alternatively, for longer term changes, the system may increase or decrease the number of receptor proteins on the surface of the target neurons. Thus, when nicotine is present for a long period in the brain, the number of nicotinic receptors for acetylcholine is altered. This type of change in receptor numbers takes much longer to reverse when the drug is removed than do the short-term modifications. Similar changes occur in receptor numbers in response to alcohol, as discussed below. As a result, researchers studying these neurochemical phenomena at various time points might obtain entirely different, although equally correct, explanations for the adaptations that accompany tolerance, whether to alcohol or to other drugs.

As might be expected, the complexities involved in this process have given rise to disagreements about which neurochemical changes are most important in alcohol tolerance, dependence, and withdrawal. Some consensus has been reached, however. This article examines postulated neuroadaptations in three systems: (1) the gamma-aminobutyric acid (GABA) neurotransmitter system; (2) the glutamate neurotransmitter system; and (3) the calcium channel system, which regulates various processes inside neurons. The focus on these three mechanisms is not intended to discount others that may prove equally or more important for AW. Even the limited focus on these few systems allows several important points about clinical relevance to be made.

## Animal Models of Alcohol Withdrawal

Meaningful research on neuroadaptation underlying AW became possible only in the early 1970’s, when investigators developed models of physiological dependence and withdrawal in laboratory animals (for example, see Goldstein and Pal 1971). Before this development, most preclinical research focused on the alcohol “preference” of laboratory rodents that consumed alcohol voluntarily. Because of the limited intake of alcohol by most rodents and the rapid elimination of alcohol from their bloodstreams, such models rarely produced a recognizable physical withdrawal syndrome. Consequently, any neurochemical findings obtained could not be related to withdrawal severity or duration. The key to success in the development of research models for withdrawal proved to be the use of “forced” alcohol administration. For example, some common models allow animals access only to liquid diets containing alcohol, whereas other models maintain continuous intoxication by keeping animals in an atmosphere containing alcohol vapor.

Those animal models clearly do not reflect all aspects of human alcohol dependence. An obvious deficiency is that the neurochemical effects of self-administration of alcohol, such as changes in forebrain dopamine release (Weiss et al. 1996), are relatively ignored. However, the forced-administration models have provided valuable data on the neurochemical changes that accompany the induction of physiological dependence on alcohol. In turn, many of the alterations in brain chemistry provide fairly convincing explanations for the physical changes that occur during withdrawal (Samson and Harris 1992).

## Neuroadaptation in the Gaba Neurotransmitter System

**Figure 2:**
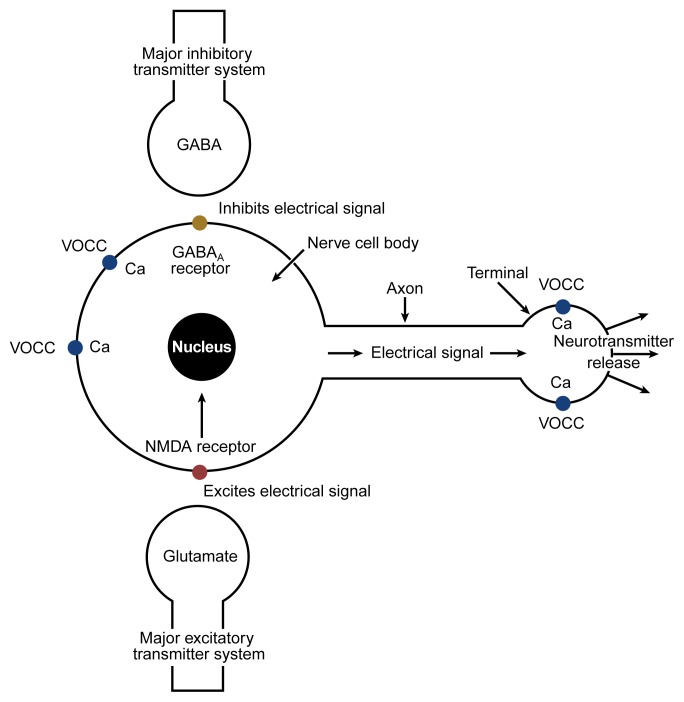
Schematic representation of some of the major neurochemical systems affected by alcohol. Nerve cells (i.e., neurons) convert chemical messages received at the cell body (at left in this simplified neuron) into an electrical signal that is conducted along the axon to the terminal (at right). At the terminal, the electrical signal is converted back into a chemical message (i.e., a neurotransmitter) that is released from the terminal and carries the information to the next neuron in the circuit. Alcohol increases (i.e., potentiates) the effects of the major inhibitory neurotransmitter in the effects tend to inhibit electrical signaling through the neuron. Alcohol further decreases electrical activity by inhibiting the major excitatory neurotransmitter, glutamate, particularly at a glutamate-receptor protein called the *N*-methyl-d-aspartate (NMDA) receptor. By inhibiting glutamate at the NMDA receptor, alcohol slows the flow of calcium (Ca) into cells. Regulation of the cell’s calcium balance is essential for normal cell function. In addition to its effects at the NMDA receptor, alcohol can alter the flow of calcium through voltage-operated calcium channels (VOCC’s) at the cell body as well as at the terminal, where calcium is necessary for neurotransmitter release.

When neurons in the brain release GABA, this neurotransmitter delivers its chemical message by combining with specific receptor proteins for GABA on the surfaces of other neurons. GABA’s effect is inhibitory; that is, it reduces the electrical activity of the target neurons. When alcohol is introduced into this system, its immediate (i.e., acute) effect is to add to (i.e., potentiate) the inhibition caused by GABA, primarily on a particular receptor protein designated “GABA_A_” (see figure 2). Consequently, neurons receiving messages through GABA are even more inhibited by this transmitter than usual when alcohol is present in the brain.

**Figure 3:**
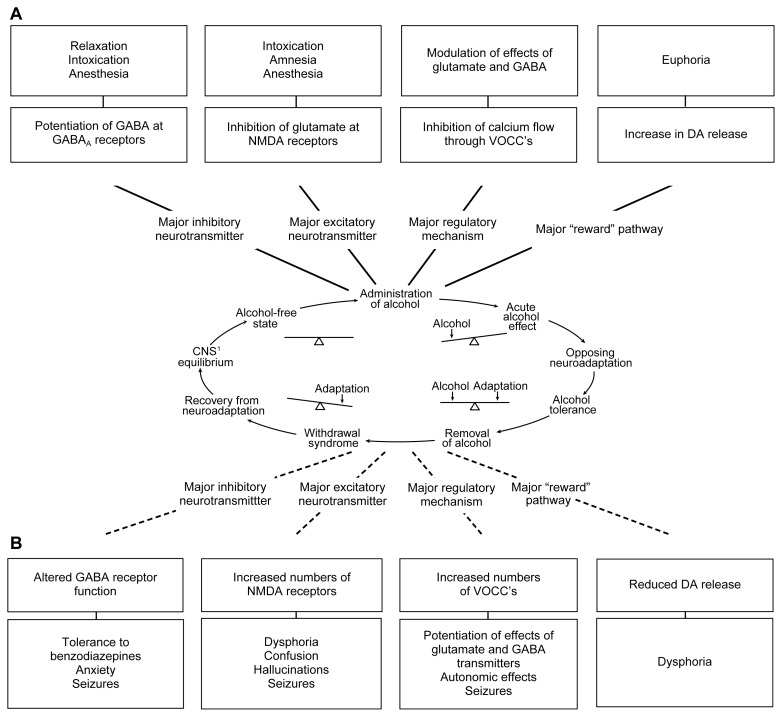
Effects of alcohol and possible relevance to dependence and withdrawal. (A) The major acute actions of alcohol and their possible relation to the behavioral consequences of drinking alcohol are shown in relation to the Himmelsbach glutamate at *N*-methyl-d-aspartate (NMDA) receptors, and inhibition of voltage-operated calcium channels (VOCC’s) may underlie the relaxation, intoxication, anesthesia, and amnesia caused by alcohol. Alcohol also increases release of the neurotransmitter dopamine (DA) in a specific area of the brain, the nucleus accumbens. This action of alcohol is not very well understood but may play an important role in the rewarding effects of drinking, such as euphoria. (B) Examples of the adaptive changes thought to oppose the acute effects of alcohol. The bottom panels show possible consequences of these adaptations during withdrawal. For example, the adaptive changes in GABA receptor proteins caused by alcohol may make benzodiazepine tranquilizers, which also act on GABA receptors, less effective (i.e., may produce tolerance). A reduction in DA release in the nucleus accumbens may accompany alcohol withdrawal and may contribute to depression, anxiety, and emotional discomfort (i.e., dysphoria), perhaps leading an alcoholic to resume his or her drinking. ^1^CNS = central nevous system.

Because GABA neurons are widespread, alcohol probably potentiates inhibition of neuronal activity in several areas of the brain. The effect is similar to that of the benzodiazepine-tranquilizing drugs, which also potentiate the action of GABA at GABA_A_ receptors, and helps explain why both alcohol and benzodiazepines are effective antianxiety agents (see figure 3).

Because alcohol increases the effects of GABA_A_ receptor activation, the Himmelsbach concept of adaptation predicts that in the long-term presence of alcohol, neurons will respond by reducing the number of GABA_A_ receptor proteins on their surfaces. This reduction should “balance” the acute effects of alcohol and produce the consequences (i.e., tolerance, dependence, and withdrawal) mentioned earlier. However, GABA_A_ receptors in the brain appear to be altered in a much more subtle way in response to alcohol. Several smaller proteins make up the GABA_A_ receptor protein, and by shuffling the way in which these subunits are arranged, neurons may be able to produce new GABA_A_ receptors that are affected very little by alcohol (Samson and Harris 1992; Littleton and Little 1994). A common research finding is that one of the small proteins, designated the alpha 1 subunit (or α_1_), decreases in number (i.e., is downregulated) after chronic exposure to alcohol, although even this reduction does not occur in all areas of the brain (Matthews et al. 1998). The downregulation of α_1_ subunits may be in keeping with the Himmelsbach concept of adaptation. Criswell and colleagues (1995) suggested that GABA_A_ receptor proteins which contain the α_1_ subunit are the most susceptible to the acute effects of alcohol. By reducing the number of receptors with an α_1_ subunit, neurons may become less responsive to the effects of alcohol, a change that could represent a potential mechanism for adaptation within the GABA system.

Although most researchers consider this alteration in GABA_A_ receptors to brain, gamma-aminobutyric acid (GABA), at the GABA_A_ receptor. GABA’s be involved in tolerance, it is not clear whether the change plays an important role in AW. To have a role in withdrawal, the adaptive mechanism (i.e., the reorganization in GABA_A_ receptor subunits) must cause a change in GABA_A_ receptor function when alcohol is no longer present. This implies a reduction in the effects of GABA itself on the function of the receptor. Investigators disagree over whether this is the case. For example, using rats, Whittington and colleagues (1995) found no evidence for underactivity of GABA_A_ receptors in an area of the brain associated with AW (i.e., the hippocampus). Similar experiments using a different regime of alcohol administration, however, produced clear evidence in favor of reduced sensitivity of GABA_A_ in the same brain area (Kang et al. 1996). Regardless of these disparate findings, the alteration in GABA_A_ receptors is a potential cause of several aspects of the AW syndrome, including anxiety and seizures (see figure 3).

Despite scientists’ uncertainty over the role of GABA_A_ receptors in AW syndrome, clinicians commonly use drugs that act on GABA_A_ receptors (e.g., the benzodiazepine diazepam) to suppress the signs of AW (Romach and Sellers 1991). This “detoxification” treatment usually succeeds in allowing someone who is physiologically dependent on alcohol to avoid seizures and other AW symptoms. Evidence suggests that less obvious harmful effects of withdrawal might still occur, however, and alternative approaches to treatment might reduce this possibility.

## Neuroadaptation in the Glutamate Transmitter System

As the most common excitatory neurotransmitter in the human brain, glutamate increases the electrical activity of neurons. This neuronal activation occurs through several different types of receptor proteins. Although alcohol inhibits many of these receptors, one specific type, the *N*-methyl-d-aspartate (i.e., NMDA) receptor,^3^ appears to be most sensitive to alcohol’s effects (Samson and Harris 1992).

Because acute alcohol administration inhibits NMDA receptors, the Himmelsbach concept predicts that neurons will compensate by producing more NMDA receptors. Several studies have indeed reported an increase (i.e., upregulation) in the number of NMDA receptors in the brain in response to alcohol (Samson and Harris 1992). Researchers theorize that neurons accomplish receptor upregulation by somehow “sensing” that their NMDA receptors are no longer being activated to the normal extent when alcohol is continuously present in the brain. This message is sent to the nucleus of the neuron, where it causes the genes specifically responsible for producing the NMDA receptor proteins to increase their activity (i.e., their expression). Enhanced gene expression results in the production of more proteins and gradually leads to an increase in the number of NMDA receptors on the surface of the affected neurons. As with many other aspects of neurochemistry in AW, however, these events probably are not quite as simple as described here. Like the GABA_A_ receptors, the NMDA receptors in the brain are made up of smaller protein subunits. It recently has been suggested that some of these subunits increase in number because of an increase in gene expression, whereas other subunits may increase for a different reason (Hu et al. 1996).

The upregulation process (producing new receptors and transporting them to the cell surface) takes several days, a time course that correlates well with both the development of alcohol tolerance and the emergence of a susceptibility to withdrawal upon alcohol cessation. The alcohol-induced increase in NMDA receptors also persists long enough after alcohol is removed to account for the duration of withdrawal. This increase in the actions of the excitatory transmitter glutamate resulting from the increased number of NMDA receptors is a potential cause of several manifestations of the AW syndrome, such as hallucinations and seizures.

In many ways the alcohol-induced alterations in the glutamate transmitter system validate the Himmelsbach scheme as it applies to the neurochemistry of alcohol tolerance and withdrawal: Initially, alcohol inhibits the actions of glutamate on the NMDA receptors; adaptation involves a straightforward homologous upregulation in the number and/or function of receptors; and withdrawal of alcohol results in hyperactivity of the glutamate system, at least partially from NMDA receptor upregulation. It remains to be seen whether the picture will stay so clear when more is learned about other adaptations that occur in the glutamate neurotransmitter system.

## Neuroadaptation in Calcium Channel Proteins

Each time a neuron is electrically activated, calcium flows into the cell through proteins called calcium channels, which are located on the outer surface of the nerve. Because these channels are opened by electrical activity, they are known as voltage-operated calcium channels (VOCC’s). Other channels (i.e., receptor-operated calcium channels, or ROCC’s) admit calcium in response to neurotransmitters attaching to receptors. The NMDA receptor is an example of this mechanism. When glutamate combines with the NMDA receptor, a channel through the protein opens and allows calcium to enter the neuron. When this entry of calcium through VOCC’s and ROCC’s occurs in the “body” of the neuron (see figure 2), the calcium entering the nerve regulates many important processes, probably including the expression of the genes that produce the NMDA receptor, GABA_A_ receptor proteins, and the proteins that form VOCC’s. At the other end of the nerve cell (i.e., the terminal), the entry of calcium ions causes the neuron to release stored neurotransmitters. Because neurotransmission is the main function of all neurons (i.e., it is the way in which they communicate), anything that interferes with calcium channel function has important and far-reaching effects. Alcohol reduces the flow of calcium into nerves through NMDA receptors and, probably, other ROCC’s as well (Lovinger 1997). It also reduces calcium entry through VOCC’s, but in this case the mechanism is less clear.

At initial administration, alcohol’s direct effects on VOCC’s are probably small at the alcohol concentrations typically achieved in the human brain (Samson and Harris 1992). However, alcohol also has indirect effects on these calcium channels through its ability to potentiate GABA neurotransmission and inhibit glutamate neurotransmission, thereby reducing the electrical activity of nerves. The continued presence of alcohol eventually leads to an increase in the numbers of VOCC’s, presumably representing an adaptation designed to compensate for alcohol’s inhibitory effects. This upregulation perhaps results from increased gene expression (Littleton and Little 1994). Likewise, the increase in the number of VOCC’s probably follows a time course similar to the alcohol-induced upregulation in NMDA receptors, both during the development of tolerance and during withdrawal.

The increase in calcium channels, whether through upregulation of ROCC’s (like the NMDA receptor) or VOCC’s, follows the pattern predicted by the Himmelsbach scheme. Although current understanding is probably an oversimplification of these changes (e.g., several different types of VOCC’s exist that may be regulated in quite different ways), adaptation in calcium channels, like the changes in receptor proteins, is one of the established mechanisms for alcohol tolerance and withdrawal (Samson and Harris 1992; Littleton and Little 1994).

Thus, during the induction of alcohol tolerance, increased numbers of VOCC’s and NMDA receptors develop on nerves in the brain, and changes occur in the GABA_A_ receptors. These adaptive changes persist during alcohol withdrawal and are believed to contribute to the withdrawal syndrome. Some aspects of the withdrawal syndrome probably are caused by a generalized increase in neuron excitability resulting from an increase in VOCC’s (Littleton and Little 1994). Other withdrawal syndrome changes may be explained specifically by neural pathways in which excitability is controlled by either NMDA receptors or GABA_A_ receptors (Samson and Harris 1992) (see figure 3).

## Limitations of Studies of Withdrawal

The changes discussed previously that occur in the brain during long-term alcohol administration are fairly convincing neurochemical explanations for the mechanisms involved in AW. However, there are certain limitations to these studies that should not be ignored. In particular, both the methods used to evaluate the neurochemical changes themselves and the models used to mimic clinical alcohol dependence may affect the types of results obtained. These limitations do not invalidate the conclusions, but they do suggest that other mechanisms of withdrawal should be considered as well.

### Types of Neuroadaptation

There are several similarities in the types of changes in GABA_A_ receptors, NMDA receptors, and calcium channels that were suggested above to underlie AW. All of these changes represent modifications in the synthesis of nerve cell proteins, which in turn results in a slowly developing alteration in either the composition or the number of these proteins in the brain. Slow adaptations like these also are reversed relatively slowly, a phenomenon that accords with the Himmelsbach explanation of withdrawal (Littleton and Little 1994). The Himmelsbach explanation predicts that the functional imbalance in the brain which causes the withdrawal syndrome should last only as long as it takes for the adaptive mechanism to be removed (see figure 1). Adaptations that are reversed quickly cannot explain withdrawal syndromes of long duration, such as that from alcohol. Another, more pragmatic, explanation may exist, however, to explain why neurochemical researchers have concentrated on the slower types of adaptations (e.g., alterations in receptor proteins and calcium channels) in the brain: This type of change is the easiest to measure.

Most neurochemical experiments in this area involve postmortem measurements of changes in the brains of animals at various times during alcohol administration and withdrawal. Semipermanent changes in protein numbers (e.g., an increase in glutamate receptors) are much easier to measure under these conditions than are more ephemeral changes (e.g., an increase in glutamate transmitter release). The latter changes are not necessarily any less important as a mechanism for withdrawal, however. For example, during AW, dopamine release may be reduced in the area of the brain (i.e., in the nucleus accumbens) in which this transmitter is increased by alcohol acutely (Weiss et al. 1996). Although short-lived changes in transmitter release such as this are difficult to measure, the depression, anxiety, and emotional discomfort (i.e., dysphoria) that may result from the reduction in dopamine are an important psychological aspect of the withdrawal syndrome (see figure 3).

### Types of Models

Another practical limitation of the results discussed applies to the type of model of dependence most commonly used. Most researchers strive to induce physiological dependence in laboratory rodents as rapidly as possible, using a single continuous period of alcohol administration (24 hours per day for several days) followed by its removal (e.g., Goldstein and Pal 1971). This single cycle of dependence is the quickest way to produce a withdrawal syndrome. The drawback of this simple model is that the continuous administration/single-cycle model does not reflect the usual pattern of alcohol self-administration in humans, which is much more episodic, with periods of voluntary abstinence (Ballenger and Post 1978).

This is important because different patterns of alcohol administration may model for dependence. The potentiation of gamma-aminobutyric acid (GABA) at GABA_A_ receptors, inhibition of produce different types of adaptation and, consequently, different mechanisms for withdrawal. For example, the variations in the effects on the GABA_A_ receptors in the hippocampus, as previously described, came from models in which alcohol administration was either constant (Whittington et al. 1995) or intermittent (Kang et al. 1996). This does not mean that withdrawal syndrome itself will appear to be different, because different mechanisms may produce identical signs and symptoms. For example, if alcohol leads to an adaptive increase in glutamate receptors in a nerve pathway in the brain, transmission along this pathway will increase in AW, in this case because the glutamate released as a transmitter will be more effective (i.e., because of an increased number of receptors for this transmitter). Alternatively, suppose the number of glutamate receptors stays the same, but the nerves in the pathway become more excitable because they have increased the number of calcium channels on their surface. Once again, transmission through the pathway will increase, although the mechanism involved is different. The outward signs and symptoms (i.e., the behavior) associated with withdrawal would be identical for both mechanisms. However, if potential treatments are to be targeted to a particular mechanism, it is necessary to first determine the true mechanisms underlying withdrawal.

Although the changes in receptor and calcium channel proteins described previously have been found in several animal models by different investigators, this is not always the case. In fact, some models that indubitably produce physiological dependence and a recognizable withdrawal syndrome never show the expected alterations in receptors and channel proteins. An example would be the difference in the relative importance of GABA_A_ receptors for withdrawal-induced excitation in the hippocampus (Whittington et al. 1995; Kang et. al. 1996). The acid test for all of these mechanisms will be whether they are found to occur in humans. In general, the human results have not been convincing. Despite repeated postmortem studies in alcoholics, scientists have not uncovered consistent alterations in the human brain. This finding is not entirely surprising given the variability in human tissue and the confounding incidence of brain damage in many alcohol-dependent subjects.

These limitations aside, the neurochemical explanations for AW, as described previously, probably include some fundamental truths; for example, hyperexcitability of nerves in the brain occurs during AW, and alterations in the GABA and glutamate transmitter systems probably contribute to this excitability (Samson and Harris 1992). Similarly, any increase in the electrical activity of nerves that occurs during severe withdrawal is inevitably associated with increased calcium entry into nerves through calcium channel proteins (whether these channels are increased or not). Even this “minimalist” view of the research findings has some clinical implications.

## Treatment Implications

A key reason for understanding the neurochemical mechanisms underlying the withdrawal syndrome is to improve methods of treatment. The following section examines areas in which advances in neurochemistry may be particularly useful.

### Detoxification

Based on the neurochemical evidence described earlier, the signs and symptoms of AW clearly are not simply the result of a reduced stimulation of GABA_A_ receptors. Despite this fact, the most common pharmacological treatment given during withdrawal continues to be a benzodiazepine or some other sedative that acts to enhance activation of GABA_A_ receptors (Romach and Sellers 1991). In addition, the changes in GABA_A_ receptor proteins believed to confer alcohol tolerance may, in fact, reduce the effects of benzodiazepines on these receptors (see figure 3), thus explaining why relatively high doses of these drugs often are needed to suppress the withdrawal syndrome.

Nevertheless, benzodiazepines continue to be used effectively and safely in detoxification, the main goal of which is to prevent the life-threatening onset of major withdrawal signs. It is possible, however, that other harmful sequelae of withdrawal exist that are not controlled by current treatments. For example, benzodiazepines are known to suppress the convulsions that occur during AW in animals. It is unclear, however, if this class of drugs suppresses all of the increased electrical activity that occurs in the brain at this time. This suppression is important because any remaining nerve excitation could produce at least two harmful consequences: (1) nerve damage and (2) changes in the brain that make subsequent episodes of withdrawal more severe.

### Nerve Damage Associated With Alcohol Withdrawal

Alcohol use has been shown to cause brain damage, and at least some of this damage may occur during periods of AW rather than during periods of alcohol administration (Lovinger 1993). One mechanism for this damage is that excessive amounts of calcium ions enter the excited nerves, switching on processes which cause the cells to self-destruct. Several connections exist between this type of damage and the neurochemical changes associated with AW. During withdrawal, hyperexcitation of nerves occurs because of increased activity in the glutamate transmitter system. This hyperexcitation is further exacerbated by an increase in the number of NMDA receptors. These receptors, which are receptor-operated calcium channel proteins, allow calcium ions to readily enter the nerve cell. Even more calcium ions enter the excited nerves through the increased numbers of voltage-operated calcium channels on the nerve surface. Most likely, all of these mechanisms work together to make nerves vulnerable to the kind of damage resulting from overexcitation (i.e., excitotoxicity) that may occur during AW (Lovinger 1993). More extensive research is necessary, however, to determine the exact link between neuronal excitation during withdrawal and the possible development of nerve cell damage.

Although alterations in GABA_A_ receptors may play a part in the increased excitation of nerves in withdrawal, it seems unlikely that drugs acting on the GABA system, such as benzodiazepines, will be able to prevent all the excitotoxic damage taking place in nerves during withdrawal. This area is one in which findings from animal research could be immediately applied to therapeutic intervention. If experiments could show that the addition of another type of drug (such as an NMDA receptor antagonist or a calcium channel blocker) increased the efficacy of current detoxification treatments against withdrawal-induced brain damage, the findings would be highly relevant to clinical practice. As an example, recent experiments by Corso and colleagues (1998) have shown that when rats are subjected to intermittent exposure to alcohol (with frequent minor episodes of withdrawal), damage occurs in nerves within the hippocampus. The researchers administered calcium channel blockers to the animals to stop the influx of calcium into nerve cells and, in theory, the resulting cell damage. Surprisingly, although the drugs were effective against damage in some parts of the hippocampus, they were ineffective and even harmful in other parts. The contrasting results emphasize the importance of mimicking clinical situations as closely as possible. Conclusions drawn from simple cellular models might suggest that withdrawal excitation is always directly equated with excitotoxic nerve damage in withdrawal; such conclusions, however, would be an oversimplification. In addition, accumulating evidence shows that intermittent alcohol administration and repeated withdrawal can produce different neurochemical effects, as well as more intense signs of withdrawal, from those seen in the single-cycle models most often used in research. This approach is discussed in the following section.

### The Severity of Repeated Alcohol Withdrawal

The continuous administration/single-cycle models of alcohol dependence may miss an important aspect of alcohol dependence in humans: that is, repeated cycles of dependence and withdrawal may have progressively worse consequences. This outcome is not always the case experimentally; it depends on variables, such as the duration of alcohol administration and the length of time between withdrawal episodes. Perhaps the best evidence that repeated withdrawal becomes progressively worse is that the incidence and severity of seizures increases during repeated withdrawal, both in animal experiments and in humans (Becker and Littleton 1996). It also has been suggested that other aspects of withdrawal, such as anxiety, also increase progressively. This is often called kindling of withdrawal severity (Ballenger and Post 1978). The term originated from research on epilepsy, in which it was observed that small electrical stimulations (i.e., excitation) in some areas of the brain have a progressively greater effect when they are repeated. Thus, a stimulation that at first produces no observable effect may produce sufficient activation of the brain to cause convulsions when it has been repeated, for example, 20 times at daily intervals. The small stimulations “kindled” an epilepsylike seizure.

Thus, at least as far as the occurrence of seizures is concerned, a similarity appears to exist between kindling and the repetitive brain excitation that occurs with repeated episodes of AW and which may lead to seizures and other symptoms. This similarity does not mean that the mechanisms for the two phenomena are identical, however. Although several potential neurochemical mechanisms exist both for kindling and for the progressive increase in seizures in withdrawal, none has yet been accepted universally. For example, some of the mechanisms proposed to explain kindling in withdrawal involve changes in the GABA transmitter system (Kang et al. 1996). However, once again, it is unlikely that drugs which affect this system selectively will be able to completely prevent the progressive increase in withdrawal severity. Although this idea has not yet been proven, evidence does suggest that current methods of detoxification have little effect on the progression of withdrawal severity (Becker and Littleton 1996).

Clearly, important questions remain as to the best AW treatment, and the use of animal models of repeated withdrawal could be of immediate clinical relevance to address these questions. Again, if it could be shown that benzodiazepines do not fully suppress the mechanisms responsible for kindling of withdrawal severity but that an additional treatment during detoxification interrupts this progression, the finding would have an immediate impact on clinical practice. Excitotoxicity (the putative mechanism for nerve damage in withdrawal) also may be subject to kindling by repeated episodes of withdrawal (Becker and Littleton 1996). Other mechanisms of nerve cell damage that may occur during repeated withdrawal (Corso et al. 1998) also are areas for investigation of therapeutic interventions.

As evidenced by the amount of space in this review dedicated to repeated withdrawal, a return to drinking after detoxification (i.e., a relapse) is a common event. Relapse itself is linked to some aspect of withdrawal (see sidebar, pp. 22–23), but once relapse has occurred in an abstinent patient, the possibility of “reinstatement” of dependence must be considered. It is worth considering whether the neurochemical mechanisms behind reinstatement of tolerance, dependence, and withdrawal are the same mechanisms that operate during the first episode of alcohol exposure.

**Relapse Prevention**

A key therapeutic challenge in treating alcohol dependence lies in finding ways of preventing relapse. Yet because relapse is a highly complicated behavior to model, much of the emphasis in neurochemical research has been on the physical withdrawal syndrome. However, some of the findings from withdrawal research may help address questions about relapse. For example, the depression, anxiety, and emotional discomfort (i.e., dysphoria) that occurs during withdrawal can occur during extended periods of abstinence and often is considered a precipitant of relapse (Connors et al. 1996).

These feelings appear to be triggered by exposure to stimuli (i.e., cues) that previously were associated with drinking alcohol (e.g., the smell of alcohol, a bar environment, or being offered an alcoholic drink). The cues trigger adaptive changes in the brain designed to oppose the effects of alcohol. If the patient does not immediately drink alcohol, then these adaptations “overbalance” the neurochemistry of the brain in much the same way as withdrawal from alcohol affects the dependent brain (for a review, see Connors et al. 1996). As a result, the patient experiences pseudo-withdrawal symptoms without ever taking a drink. Faced with these symptoms, the patient then may relapse to alcohol consumption.

As an example, suppose an alcohol-dependent person who is trying to maintain abstinence is offered an alcoholic drink. In the past, this cue always would have preceded acceptance of the drink and the arrival of alcohol in the brain; consequently, the cue is strongly associated with the neuroadaptation to alcohol (see figure). In response, the person becomes highly dysphoric and may even start sweating and shaking, all of which are symptoms similar to those that occur during the early stages of alcohol withdrawal. These symptoms may be caused by the cue-induced initiation of neuroadaptation intended to oppose alcohol. Based on past experience, the person knows that these very unpleasant sensations can be relieved only by drinking alcohol. If he or she does accept the drink, further neuroadaptation will occur in the brain. To offset these alterations, the person may drink even more alcohol; consequently, relapse will be well under way.

Although similarities exist among the consequences of cue-induced relapse and alcohol withdrawal, the adaptations responsible for each are unlikely to be identical. Those adaptations thought to underlie alcohol withdrawal involve chemical alterations, such as increases in receptor protein production, which are slow to develop, usually requiring several days. To precipitate relapse, however, conditioned adaptation must be rapid, beginning within minutes of cue exposure. Alterations in electrical activity in specific circuits in the brain are a more likely explanation for such rapid changes.

If similarities do exist between withdrawal and cue-induced craving, they probably occur in the neurotransmitters involved in the two processes (e.g., the glutamate and gamma-aminobutyric acid [GABA] systems). Such similarities might be especially useful for developing more effective treatments. For example, the new anticraving drug called acamprosate—which has recently undergone several successful clinical trials for relapse prevention in Europe—inhibits the *N*-methyl-d-aspartate (NMDA) receptor and has been found to reduce some nonphysical aspects of alcohol withdrawal in animal models (Spanagel and Ziegelgansberger 1997). Acamprosate’s actions also may prove useful for inhibiting the cue-induced withdrawallike symptoms that lead to relapse. Further research is needed to determine the exact mechanism underlying acamprosate’s effects. Still, this example represents yet another area in which basic research may prove highly useful for solving the major clinical problem of relapse to drinking.—***John Littleton***

**Figure:**
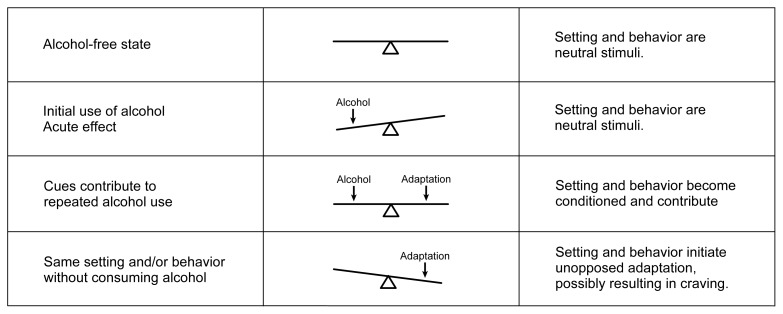
The seesaw analogy for neuroadaptation applied to conditioning and craving. The top row shows brain neurochemistry in an alcohol-free state. At this time the environment in which alcohol is consumed (i.e., the setting) and the behavior involved in drinking are neutral stimuli; that is, they have no association with drinking and no special impact on the brain. The same is true of the initial use of alcohol (second row), in which the brain’s neurochemistry is unbalanced by alcohol’s actions. If the drug is taken repeatedly in the same setting and in the same way, however, these stimuli become capable of eliciting neuroadaptations similar to those elicited by alcohol itself (third row). Exposure to such conditioned stimuli in the absence of alcohol may thus lead to unopposed neuroadaptation, which potentially may be expressed as a craving for alcohol.

ReferencesConnorsGJMaistoSADonovanDMConceptualizations of relapse: A summary of psychological and psychobiological modelsAddiction91SupplS5S1319968997777SpanagelRZiegelgansbergerWAnti-craving compounds for ethanol: New pharmacological tools to study addictive processesTrends in Pharmacological Science18545919979090311

### Reinstatement

Once relapse occurs, the general clinical consensus is that physiological dependence is reinstated rapidly (Ballenger and Post 1978). In other words, only a relatively short period of drinking is necessary before tolerance develops and the effects of withdrawal from alcohol again become severe. There are several potential neurochemical explanations as to why reinstatement should be so rapid, but to date no real evidence for or against these explanations exists. Only one of these mechanisms is discussed here, and it illustrates the deficiencies in current understanding.

The proteins (e.g., NMDA receptors and calcium channels) that increase in number during the induction of dependence rapidly decrease during withdrawal. The speed with which these potentially damaging proteins disappear (Samson and Harris 1992) implies that they must be actively removed from the nerve cell surface during withdrawal. In similar conditions, when proteins are removed they rarely are immediately destroyed, however. Instead they are removed from the nerve cell surface and sequestered inside the cell. Then, if the proteins are required again, they can be recycled back to the nerve cell surface. This recycling may be what takes place when a patient relapses to drinking soon after detoxification. The adaptations necessary to oppose alcohol’s effects already are present in nerve cells in the brain; the proteins need only be recycled (or reassembled) to reinstate alcohol tolerance and dependence. Recycling may explain why the onset of physiological dependence is so much more rapid the second time around. It also may mean that the mechanism for reinstatement of dependence differs subtly from the mechanisms that neuroscientists have uncovered in the common single-cycle models of dependence and withdrawal.

In other words, in the single-cycle models, the neurochemical mechanisms are developed “from scratch,” implying that the changes that occur in the production of receptor and ion channel proteins take place slowly. In reinstatement the same end results are achieved, but the mechanisms already exist and thus come into play much more rapidly. Treatments aimed at slow changes (in gene expression, for example), which would be effective against the primary induction of dependence, might be useless against reinstatement of dependence. The mechanisms behind the reinstatement of dependence are important therapeutic targets but they have not been extensively studied. This statement is not intended as a criticism, however; without a thorough understanding of the simple, single-cycle models, none of this speculation on mechanisms for reinstatement would be possible. Nevertheless, now may be the time to reevaluate the models of dependence and withdrawal in relation to the most important clinical objectives.

## Conclusion

Since the first introduction of appropriate animal models for AW, our understanding of the neurochemical mechanisms involved in this process has increased dramatically. Findings from this field of neuroscience represent one of the success stories in alcohol research. The disappointing aspect of this research is that advances in understanding have had relatively little impact on clinical treatment of alcohol dependence or withdrawal. As stated earlier, however, the situation is now changing. As basic science begins to address questions more directly relevant to the major clinical problems, findings in neurochemistry and neuropharmacology will be translated into advances in treatment. The therapeutic problems of minimizing long-term consequences of detoxification, the prevention of relapse, and the delay of reinstatement are all examples that will benefit from neurochemical study. The experiments required are not as straightforward as those that have taken place over the past 25 years, but eventually they should have a greater clinical impact.

## Footnotes

1 Nerve cells (or neurons) are the key players in the activity of the nervous system. In general, they signal to each other by releasing neurotransmitters. For more information on the principles of nerve cell communication and research into the neurochemistry of alcohol addiction, please consult the recent neuroscience issue of *Alcohol Health & Research World* (Vol. 21, No. 2, 1997).

2 Neurotransmitters produce their effects on the neurons receiving their message by combining with specific receptor proteins found on the surfaces of these cells.
